# Alginate-Capped Silver Nanoparticles as a Potent Anti-mycobacterial Agent Against *Mycobacterium tuberculosis*

**DOI:** 10.3389/fphar.2021.746496

**Published:** 2021-11-17

**Authors:** Cheng-Cheung Chen, Yih-Yuan Chen, Chang-Ching Yeh, Chia-Wei Hsu, Shang-Jie Yu, Chih-Hao Hsu, Ting-Chun Wei, Sin-Ni Ho, Pei-Chu Tsai, Yung-Deng Song, Hui-Ju Yen, Xin-An Chen, Jenn-Jong Young, Chuan-Chung Chuang, Horng-Yunn Dou

**Affiliations:** ^1^ Institute of Preventive Medicine, National Defense Medical Center, Taipei, Taiwan; ^2^ Graduate Institute of Medical Science, National Defense Medical Center, Taipei, Taiwan; ^3^ Department of Biochemical Science and Technology, National Chiayi University, Chia-Yi, Taiwan; ^4^ National Institute of Infectious Diseases and Vaccinology, National Health Research Institutes, Zhunan, Taiwan; ^5^ School of Pharmacy, National Defense Medical Center, Taipei, Taiwan; ^6^ School of Dentistry and Graduate Institute of Dental Science, National Defense Medical Center, Taipei, Taiwan; ^7^ Department of Dentistry, Tri-Service General Hospital, Taipei, Taiwan; ^8^ Department of Biological Science and Technology, National Yang Ming Chiao Tung University, Hsinchu, Taiwan

**Keywords:** mycobacterium tubeculosis, antimycobacterial agent, MDR TB, dormant TB, silver nanopaiticles

## Abstract

Tuberculosis (TB) is a leading cause of death from a single infectious agent, *Mycobacterium tuberculosis* (*Mtb*). Although progress has been made in TB control, still about 10 million people worldwide develop TB annually and 1.5 million die of the disease. The rapid emergence of aggressive, drug-resistant strains and latent infections have caused TB to remain a global health challenge. TB treatments are lengthy and their side effects lead to poor patient compliance, which in turn has contributed to the drug resistance and exacerbated the TB epidemic. The relatively low output of newly approved antibiotics has spurred research interest toward alternative antibacterial molecules such as silver nanoparticles (AgNPs). In the present study, we use the natural biopolymer alginate to serve as a stabilizer and/or reductant to green synthesize AgNPs, which improves their biocompatibility and avoids the use of toxic chemicals. The average size of the alginate-capped AgNPs (ALG-AgNPs) was characterized as nanoscale, and the particles were round in shape. Drug susceptibility tests showed that these ALG-AgNPs are effective against both drug-resistant *Mtb* strains and dormant *Mtb*. A bacterial cell-wall permeability assay showed that the anti-mycobacterial action of ALG-AgNPs is mediated through an increase in cell-wall permeability. Notably, the anti-mycobacterial potential of ALG-AgNPs was effective in both zebrafish and mouse TB animal models *in vivo*. These results suggest that ALG-AgNPs could provide a new therapeutic option to overcome the difficulties of current TB treatments.

## Introduction

Tuberculosis (TB) is a contagious disease caused by *Mycobacterium tuberculosis* (*Mtb*) and remains the leading cause of mortality among infectious diseases worldwide. Despite global health efforts, TB is difficult to eradicate owing to the lack of an effective preventive vaccine, a cumbersome treatment regimen, and the emergence of drug-resistant *Mtb* strains. *Mtb* that is resistant to at least isoniazid (INH) and rifampicin (RIF) is defined as multidrug-resistant tuberculosis (MDR-TB), whereas resistance to INH, RIF, any fluoroquinolone, and at least one of three injectable second-line drugs (i.e., amikacin, kanamycin, or capreomycin) is defined as extensively drug-resistant tuberculosis (XDR-TB) (Seung et al., 2015; Prasanna and Niranjan, 2019). Although four or five second-line anti-TB drugs are usually employed as part of the regimen to treat MDR-TB and XDR-TB, these agents target only a small number of cellular processes for inhibition of *Mtb*, namely protein translation, ATP synthesis, lipid catabolism, and transport, which may give rise to cross-resistance during long-term therapy (Alzahabi et al., 2020). The current regimens for drug-susceptible and drug-resistant TB are 6 months and >18 months, respectively. The lengthy, complex regimen and toxic side effects can result in partial non-compliance that may cause either treatment failure or the emergence of new drug resistance. Thus, there is an unmet need for developing novel therapeutics and improved intervention against TB.

*Mtb* is mainly an intracellular pathogen, and once inhaled into the lungs the mycobacteria are engulfed by macrophages (Guirado et al., 2013). After establishment of the primary infection, TB will develop into a latent infection by thwarting inflammatory responses and escaping from immune clearance (Guirado et al., 2013; Zhai et al., 2019). It is estimated that one-third of the world’s population is latently infected with *Mtb* (Gideon and Flynn, 2011), of which approximately 90–95% are asymptomatic. Thus, latent tuberculosis infection (LTBI) is a major characteristic of *Mtb* bacilli*,* which can turn off many vital metabolic activities and enter a dormant state when confronted with oxygen and nutrient insufficiency and the acidic environment of host macrophages. Small heat shock proteins (sHSPs) are known to serve as molecular chaperones of *Mtb* polypeptides (de Jong et al., 1998) and are typically produced in response to mild heat shock or other stresses (Lindquist and Craig, 1988). sHSP16.3 (a 16.3 kDa protein) is a stress-induced protein (but not heat-induced) encoded by the *hspX* gene of *Mtb* and plays an important role in the survival of the *Mtb* bacterium during dormancy (Jee et al., 2018). Deletion of *hspX* led to an increase in *Mtb* growth in infected mice whereas its overexpression reduced the expansion of bacilli in the early course of infection (Hu et al., 2006). Expression of *hspX* drives cell-wall-thickening of the *bacillus*, thus indirectly promoting dormancy, since the thickened cell wall helps the bacilli counteract the host’s immune defenses during the early phase of infection (Batt et al., 2020). Moreover, the dormant bacilli in patients with LTBI become much less susceptible to antibiotics compared to bacilli with normal metabolic activity, again because of the thickened cell wall (Dutta and Karakousis, 2014). The emergence of drug-resistant strains of mycobacteria has caused TB to become hard to treat. Improved therapeutic approaches are needed to overcome the limitations of the current regimens. The hydrophobic cell wall of mycobacteria reduces the ability of anti-TB drugs to penetrate the cell and interact with their respective molecular targets (Smith et al., 2013; Vilchèze, 2020). Accordingly, it is of utmost importance to develop drugs that can react directly with the cell wall of mycobacteria and disrupt its integrity. The limited choices of newly approved antibiotics have sparked the current research interest in alternative antibacterial molecules such as nanotechnology and advanced materials (Kinhikar et al., 2010; Beyth et al., 2015; Wang et al., 2017a; Wang et al., 2020a). In recent years, silver nanoparticles (AgNPs) have received much attention in bioengineering and biomedicine for disparate uses as catalysts, biosensors, and anti-microbial and anti-tumor drugs (Zhang et al., 2016; Bondarenko et al., 2018; Farooq et al., 2019; Tăbăran et al., 2020; Yin et al., 2020). AgNPs work as anti-bacterial agents through a variety of mechanisms (Dakal et al., 2016; Yin et al., 2020) which include disruption of bacterial membranes and cell walls to trigger cell leakage, and reduction in the levels of antioxidants leading to reduction-oxidation (redox) imbalance and oxidative damage to bacterial DNA (Dakal et al., 2016). AgNPs for use in anti-TB therapy have the potential to increase the susceptibility of drug-resistant *Mtb* strains which are tolerant to most of the organic antibiotics currently in use (Tăbăran et al., 2020). Although AgNPs provide good anti-microbial activity mediated by physicochemical mechanisms and may prove to be effective in improving TB therapy, several critical therapeutic issues still need to be overcome, notably poor drug delivery, inconsistent intra-macrophagic anti-mycobacterial effectiveness, and off-target toxicity (AshaRani et al., 2009; Kemp et al., 2009; Liao et al., 2019; Ferdous and Nemmar, 2020).

The synthesis of AgNPs by nanotechnologies can be through different approaches, including physical and chemical methods. Physical methods use a tube furnace at atmospheric pressure to evaporate and condense nanoparticles. Nevertheless, the downsides of physical methods are low yield and high energy consumption. Conventional chemical methods, including the use of chemical reductants and polymers, have been used for the reduction and stabilization of AgNPs. but chemical methods risk solvent contamination that increases cytotoxicity and biohazard potential, and can also impact particle consistency (Zhang et al., 2016). Chemical reductants like sodium borohydride, hydrazine, and dimethylformamide are required for reduction of the silver salt during synthesis of AgNPs (Nam et al., 2016; Zhang et al., 2016; Shao et al., 2018), and polymers such as polyaniline and polyvinylpyrrolidone have been adopted to prevent aggregation of the nanoparticles during the synthetic process (Wang et al., 2005; Sharma et al., 2012). However, these chemicals are generally associated with biological side effects and environmental impacts owing to their cytotoxicity and low biocompatibility. To address these limitations, we propose using an environmentally and biologically friendly material to synthesize AgNPs.

The nanostructured architectures of various simple marine organisms such as algae, diatoms and sponges facilitate high metal uptake suitable for the synthesis of metallic nanoparticles, and thus offer a biological approach to their synthesis (Soltmann et al., 2010). Since seaweeds and many algae originate from marine environments, they are relatively easy and inexpensive to obtain. In addition, the natural polysaccharide alginate, has already been used for delivery of anti-cancer drugs, insulin, indomethacin, and anti-TB drugs (Huang et al., 2012; Nasiruddin et al., 2017; Hariyadi and Islam, 2020) because of its favorable and diverse properties. Alginate is a water-soluble, poly-anionic linear carbohydrate composed of 1,4-linked *α*-*L*-guluronic and *ß*-*D*-mannuronic acid polymeric residues. Moreover, it is inexpensive, biocompatible and available as a nonhazardous sodium salt. The applications of alginate in the biotechnology industry include its use as a thickening and gelling agent in foods, as an emulsifier and colloidal stabilizer for drug delivery, and as a biodegradable wound dressing which maintains a moist environment over the wound (Wang et al., 2017b; Beaumont et al., 2021; Chudasama et al., 2021). In the present study, we tested alginate as a nontoxic stabilizing and/or reducing agent for green synthesis of AgNPs in aqueous solution. By the biocompatibility properties of alginate with the excellent sterilizing ability of AgNPs, we hoped to create an improved AgNP drug for use in TB treatment, including drug-resistant strains. The aim was to investigate the biocidal effect of alginate-capped AgNPs (ALG-AgNPs) on active and latent TB caused by mycobacterial infection.

## Materials and Methods

### Drugs and Chemicals

Sodium alginate (ALG, from brown algae, viscosity = 10 cps in 1% H_2_O at 25°C, M/G ratio = 3.42), rifampicin (RIF) and isoniazid (INH) were purchased from Sigma (St. Louis, MO, United States). Silver nitrate, D (+)-glucose, and sodium hydroxide were purchased from Merck (Darmstadt, Germany).

Green synthesis of ALG-capped silver nanoparticles (ALG-AgNPs).

ALG-AgNPs were synthesized according to our previous reports (Chang et al., 2017; Young et al., 2018) by the reduction of Ag^+^ ions in an alkaline aqueous medium using glucose as a reducing agent and ALG as a stabilizing and/or reducing agent. Briefly, an aqueous solution of silver nitrate was added dropwise to an aqueous solution of ALG, glucose, and NaOH at room temperature while stirring. After the reaction, the mixture was diluted 50-fold to measure the surface plasmon resonance (SPR), size, and zeta potential. The excess base and reactants remaining in the suspension were dialyzed against water using a Dialysis Cassette (Slide-A-Lyzer®, G2, 2000 MWCO) until the pH of the aqueous solution became neutral. Table 1 summarizes the conditions used to synthesize the ALG-AgNPs, and the particle size and zeta potential of the as-prepared and dialyzed colloidal solution. The purified ALG-AgNPs were then subjected to different spectral measurements, TEM imaging, and *in vitro* or *in vivo* tests. The dialyzed aqueous solutions of ALG-AgNPs were stable for more than 6 months at 4°C.

**TABLE 1:** The conditions used to synthesize alginate-capped AgNPs as well as particle size, zeta potential (ζ).

Final concentration				As-prepared		Dialyzed	
AgNO_3_ (mM)	ALG (mg/ml)	Glu (mM)	NaOH (mM)	Size (nm)	Zeta (mV)	Size (nm)	Zeta (mV)
5	5	2.5	40	65.5 ± 18.1	−50.3 ± 1.9	69.7 ± 18.4	−47.0 ± 12.2

*Measured by ICP-Mass.

### Characterization of ALG-AgNPs

SPR values were measured using UV–vis absorption spectra on a Perkin Elmer Lambda 35 spectrophotometer (PerkinElmer, Waltham, MA, United States), scanning from 300 to 700 nm. Samples were run in disposable 1.5 ml plastic cuvettes. Milli-Q water was used in the reference cell. Concentrated samples (0.02 ml) were diluted 50-fold with 0.98 ml of Milli-Q water before detection.

The particle size was determined by photon correlation spectroscopy (Zetasizer Nano-ZS; Malvern Instruments, United Kingdom). The instrument contains a 4 mW He-Ne laser that operates at a wavelength of 633 nm, and an avalanche photodiode (APD) detector. The scattered light was detected at an angle of 173°. Raw data were subsequently correlated to the mean hydrodynamic size by cumulant analysis (Z-average mean). The zeta potentials (ζ) of all ALG-AgNPs were analyzed using laser Doppler anemometry (Zetasizer Nano-ZS; Malvern Instruments, United Kingdom).

The morphology of ALG-AgNPs following purification as described previously, was examined using a JEOL JEM-1200 EXII transmission electron microscope (JEOL, Tokyo, Japan). A typical method of preparing TEM samples was as follows: one drop of the ALG-AgNP suspension was deposited on a 200-mesh Formvar/carbon-coated copper grid, and excess solution was removed by wicking with filter paper to avoid particle aggregation. The images were examined by TEM at 80 kV.

The crystalline structure of the ALG-AgNPs was examined by X-ray diffraction (XRD), which was carried out on a Bruker D2 Phaser powder diffractometer (Karlsruhe, Germany) operated with a Cu K*α* radiation source filtered with a graphite monochromator (0.154 nm). X-rays were generated at 30 kV and 10 mA. The XRD scans were recorded at 2θ from 10 to 90° at a scan rate of 0.05°/0.5 s. X-ray photoelectron spectra (XPS) were collected using a VG-Scientific ESCALAB 250 instrument. Elemental analysis was conducted using a pass energy of 20 and 160 eV for the survey. Al K*α* radiation at 1486.6 eV was used and the peak positions were calibrated internally to the C1s peaks at 284.6 eV. A colloidal sample was deposited on a silicon substrate and dried in a vacuum chamber at 110°C for XPS signal measurement. The operating power was 300 W, and the scan rate was 0.1 eV/150 ms. The XRD and XPS analysis were measured by the Precious Instrument Center of Ministry of Science and Technology of Taiwan. Colloid silver samples were lyophilzed to powder before measurement.

### Bacterial Strains and Cultures

The *Mtb* strains used in this study were H37Ra, H37Rv, W6 (Beijing strain); two multidrug-resistant (MDR) strains, KVGH376 and KVGH264, from Kaohsiung Veterans General Hospital (Kaohsiung, Taiwan); two extensively drug-resistant (XDR) strains, TCHL002, TCHL017, from Taipei City Hospital-Linsen branch (Taipei, Taiwan); and two strains from Changhua Christian Hospital (Changhua, Taiwan), CHCH005 (Beijing strain) and CHCH029 (East African-Indian strain). All strains were routinely grown at 37°C with 5% CO_2_ in 7H10 agar (Becton, Dickinson and company (BD), MD, United States) supplied with 10% oleic acid-albumin-dextrose-catalase (OADC) (Creative life sciences, Taipei, Taiwan) enrichment and 0.5% glycerol (Union Chemical works LTD., Hsinchu, Taiwan) or 7H9 broth (BD, MD, United States) supplied with 10% OADC enrichment and 0.5 mg/ml Tween 80 (Sigma, St. Louis, MO, United States).

### Minimum Inhibitory Concentration Determination and Drug Susceptibility Testing

Minimum inhibitory concentration (MIC) of the ALG-AgNPs was determined using a microplate Alamar blue assay (Franzblau et al., 1998). The Alamar blue assay was performed in sterile 96-well plates. The different *Mtb* strains in log phase were cultured in 7H9 medium supplemented with 10% OADC and cultured at 37°C with shaking at 220 rpm for 3–4 weeks before use. 1.56 × 10^5^ colony forming units (CFUs) per 100 μL of different *Mtb* strains were inoculated into 96-well plates containing 100 μL 7H9 broth with different amounts of ALG-AgNPs. Additional controls included wells with *Mtb* cells without ALG-AgNPs and wells with only ALG-AgNPs and media. After 5 days of incubation at 37°C, 50 μL of 10× Alamar blue (Invitrogen, CA, United States) with 10% Tween 80 at a 1:1 ratio was added to control wells of *Mtb* cells alone for 24 h to confirm the growth of *Mtb* cells, then 50 μL of Alamar blue mix solution was added to each well, and the plates were re-incubated for 24 h. Each well either remained blue or turned pink, representing no growth or growth, respectively. Some wells showed a violet color and were recorded as growth. The MIC value was determined as the lowest concentration of ALG-AgNPs which prevented the color change in the well. For drug susceptibility testing, cultured mycobacteria (100 μL) from the Alamar blue assay representing four *Mtb* strains [H37Rv, W6 (Beijing), KVGH264 (MDR), and TCHL017 (XDR)] were treated with PBS or the indicated concentrations of ALG-AgNPs for 0 and 7 days, then titrated for three dilutions (10×, 100× and 1000×) and performed in three independent experiments. CFU values were determined by using 100 μL bacterial mixture diluted on 7H10 agar plates. Plates were kept at 37°C for 3–4 weeks and the number of colonies on each plate was counted.

### Infection of THP-1 Cells

The human monocytic cell line THP-1 was purchased from BCRC (Bioresource Collection and Research Center, Hsinchu, Taiwan). THP-1 cells were cultured in RPMI 1640 supplemented with 10% FBS, 2 mM L-glutamine, 100 U/ml penicillin, 100 µg/ml streptomycin, and 0.05 mM 2-mercaptoethanol and incubated at 37°C under 5% CO_2_. 10^5^ THP-1 cells/well were seeded into 96-well culture plates in RMPI (10% FBS, L -glutamine, no antibiotic) with phorbol 12-myristate 13-acetate (PMA, Sigma-Aldrich) at a final concentration of 100 nM. Following 3 days of PMA stimulation, THP-1 cells differentiated into plastic-adherent, well-spread cells with macrophage-like morphology. Four strains of *Mtb* [H37Rv, W6 (Beijing), KVGH264 (MDR), and TCHL017 (XDR)] were used to infect PMA-differentiated THP-1 macrophages at an MOI of 1 for 24 h. Cells were then wash thoroughly with PBS to remove excessive extracellular bacilli. After that, PBS, 1 μg/ml rifampicin or 25, 50, 100 μg/ml ALG-AgNPs were added to the wells and then incubated for five more days. Finally, the cells were lysed in distilled water containing 0.1% SDS for 15 min at room temperature. The cell lysates from each sample were used to determine the number of CFUs on Middlebrook 7H10 agar after incubation for 21 days.

### *In vitro* Model of Mycobacterial Non-replicating Persistence Under Hypoxic Conditions

Cultures were taken from a frozen stock of *Mtb* strain H37Rv. The cells were thawed, grown to log phase (OD_600_ = 0.6–0.8) and then diluted 1:100 into a 15-ml screw-capped conical tube filled with 10 ml of Middlebrook 7H9 medium supplemented with 0.2% glycerol, 10% OADC and 0.025% Tween 80 at 37°C. The head space to volume ratio (HSR) was maintained at 0.5. The tubes were tightly sealed with Parafilm^®^ (Bemis NA). A visual indication of oxygen depletion was observed by the addition of sterile methylene blue solution (500 mg/ml) to a final concentration of 1.5 mg/ml to 10-ml standing cultures maintained under hypoxic conditions in Middlebrook 7H9 supplemented as above. Control tubes containing Middlebrook 7H9 medium and methylene blue, but no bacteria, were also set up. The decline in absorbance of methylene blue is routinely used for measuring hypoxia to confirm O_2_ depletion. In the cultivation of *Mtb*, methylene blue decolorization starts when the dissolved oxygen concentration falls below 3%. Hence, complete decolorization of methylene blue, indicative of hypoxia, was reached at 12 days. After the cultures had reached hypoxia and grown to OD_600_ = 0.5, approximately 5 × 10^7^ CFU/ml were treated with PBS, rifampicin (1 μg/ml), isoniazid (0.2 μg/ml), and different amounts of ALG-AgNPs (25, 50, and 100 μg/ml) for 5 days. Aliquots from each sample were used to determine the number of CFUs on Middlebrook 7H10 agar after 21 days.

### RNA Extraction and Real-Time Reverse Transcription-PCR

Oxygen-replete and non-replicating persistent (NRP) stage *Mtb* cultures were harvested. *Mtb* cultures exposed to hypoxia were removed from the hypoxia chamber and kept on ice with minimal disturbance for 30 min to arrest the cells at their actual metabolic state. This cell suspension was centrifuged in a pre-cooled rotor at 10,000 × g for 10 min at 4°C. Finally, the supernatant was removed, and the pellet was snap-frozen in liquid nitrogen and stored at -80°C. In the case of log phase cells from oxygen replete cultures, bacteria were harvested as per the hypoxic cultures. Total RNA was extracted by homogenizing the cells with glass beads in 1 ml Trizol reagent (Thermo Fisher Scientific, United States) on ice for 10 min, then the bacterial clumps were homogenized using a Minilys^®^ homogenizer (Bertin technologies, France) with bead-beating for 15 s 6 times at medium speed (4,000 rpm). The homogenates were left on ice for 20 min, then chloroform was added for 2–3 min. The sample was centrifuged for 15 min at 12,000 × g at 4°C. The mixture separates into a lower red phenol-chloroform organic phase, an interphase, and a colorless upper aqueous phase. The aqueous phase containing the RNA was transferred to a new tube containing 0.5 ml isopropanol for 10 min. The tube was centrifuged for 10 min at 12,000 × g at 4°C. Total RNA precipitate forms a white gel-like pellet at the bottom of the tube. The pellet was washed in 1 ml of 75% ethanol and resuspended in 20–50 μL of RNase-free water. RNA concentration and quality were measured using a Nanodrop^®^ ND-1000 spectrophotometer (Thermo Fisher Scientific, United States). The cDNA was reverse-transcribed using Moloney murine leukemia virus reverse transcriptase and random hexamer oligonucleotides for priming (Life Technologies, CA, United States). Expression of the *hspX* and *esat-6* genes was determined by real-time quantitative PCR analysis using an ABI VII7 system (Applied Biosystems, Foster City, CA, United States). Transcript levels between various RNA samples were normalized using 16S rRNA. Gene expression was quantified using the ΔΔCt method. Primers used for RT-PCR were as follows: *hspX* Fwd: 5′-GGA​AGA​CGA​GAT​GAA​AGA​GG-3′ and Rev: 5′-AAC​CGC​CAC​CGA​CAC​AGT​AAG-3′; *esat-6* Fwd: 5′-CCA​TTC​ATT​CCC​TCC​TTG​ACG-3′ and Rev: 5′-TGC​GAA​CAT​CCC​AGT​GAC​G-3′; and 16S rRNA Fwd: 5′-TTG​ACG​GTA​GGT​GGA​GAA​GAA​GC-3′ and Rev: 5′-CCT​TTG​AGT​TTT​AGC​CTT​GCG​G-3′.

### Bacterial Membrane Permeability Assay

Log phase (OD_600_ = 0.8) cells of *Mtb* strain H37Ra (8 × 10^7^ CFU/ml) were treated with 50 μg/ml ALG-AgNPs in a flask for 24 h, while untreated H37Ra cells were used as a control. The bacteria were harvested by centrifugation at 3,000 × g for 10 min and washed with 0.9% NaCl solution. Then, the bacteria were stained with 8 μg/ml propidium iodide dye (Sigma) in 0.9% NaCl solution for 15 min and kept in the dark. After staining, the bacteria were centrifuged and washed twice with 0.9% NaCl solution. The pellets were resuspended in 0.9% NaCl solution. Bacteria with damaged membranes appeared red when visualized by fluorescence microscopy, whereas intact bacterial cells were not stained.

### Zebrafish Embryo—*M. marinum* Infection Model

The mycobacterial shuttle vector pMV261-DsRed was constructed and electroporated into *Mycobacterium marinum* (*M. marinum*) cells. Transformants were selected on 7H10 plates supplemented with 50 μg/ml kanamycin. The AB/TL wild-type zebrafish embryos and larvae used for these experiments were obtained from a breeding stock of adult animals kept in our zebrafish core facility (NHRI, Taiwan). Embryos and larvae were maintained in fish water (FW) at 28.5°C from collection and throughout the experiments. The larvae were transferred to FW containing 0.2 mM 1-phenyl-2-thiourea (PTU) at 24 h post fertilization (hpf). The larvae were maintained in the FW with PTU for the duration of the experiment to prevent pigment formation and maintain optical transparency. The larvae were dechorionated within 26–28 hpf. Larvae were anesthetized by tricaine in FW for 5–10 min to allow infection by microinjection of *M. marinum*-DsRed *via* the caudal vein. Prior to microinjection, *M. marinum*-DsRed cells were grown to log phase, OD_600_ = 0.7–0.8 ( ≑ 7–8 × 10^7^ CFU/ml), then 4.6 nL (322–368 CFU/larvae) were microinjected into the caudal vein per zebrafish larvae. The infected larvae were treated with PBS, 100 μg/ml rifampicin or 200 μg/ml ALG-AgNPs for 5 days (the medium was changed every 2 day). For observation of bacterial infection, five fish per group were inoculated for each experiment and two experiments were performed. At 5 days post infection (dpi), bacterial infection was observed with a fluorescence microscope. Bright field and fluorescence images were recorded and analyzed by ImageJ software. After observation of bacterial infection, five zebrafish larvae were pooled together and lysed in 1 ml PBS containing 0.1% SDS. The homogenates were diluted at 10 × and 100 × in triplicate and plated on 7H10 agar plates. The bacterial burden was analyzed by CFU assay, and two experiments were performed. All procedures involving zebrafish embryos were performed in compliance with NIH guidelines for the use and care of laboratory animals and approved by the Institutional Animal Care and Use Committee of the National Health Research Institutes, Taiwan (#NHRI-IACUC-106123, 10/15/2018).

### Maximum Tolerated Dose Test and the Mouse Model of Mycobacterial Infection

Female BALB/c mice 6–8 weeks old were purchased from the National Laboratory Animal Center (Taipei, Taiwan). All mice were kept in individually ventilated cages at the Animal Center of the National Health Research Institutes (Miaoli, Taiwan). BALB/c mice were initially administered with 10, 50, 100 mg/kg of ALG-AgNPs by oral gavage or intravenous injection for the sighting study. Before the start of the toxicity tests, mice were fasted for 3–4 h. Following the period of fasting, the mice were weighed, and the fixed doses of ALG-AgNPs administered. During this period, mice were observed daily for at least 14 days without clear signs of toxicity. As there was no evident toxicity or mortality, the mice were further dosed at 500 mg/kg ALG-AgNPs by oral gavage or 250 mg/kg by intravenous injection once daily for 2 weeks. Mice were observed post-administration at 4 and 6 h, and then monitored daily for body weight and survival for 2 weeks. For determining the efficacy of ALG-AgNPs in the TB mouse model, BALB/c mice were infected with 10^6^ CFU of *Mtb* strain H37Ra *via* intravenous injection. The treatments started at 2 weeks post-infection (day 14) and the mice were given intravenous injections of PBS (control) or different amounts of ALG-AgNPs (10, 50 mg/kg) and rifampicin (10 mg/kg) 5 days per week for 2 weeks (from day 14 to day 28). After euthanasia, the lungs were collected, homogenized and plated onto 7H10 agar at appropriate dilutions. The efficacy of the ALG-AgNP treatments was determined by the CFU of H37Ra in the lungs. All procedures were performed and approved by the Institutional Animal Care and Use Committee of the National Health Research Institutes, Taiwan (#NHRI-IACUC-108077, 04/01/2019).

### Lactate Dehydrogenase (LDH) Cytotoxicity Assay

Cytotoxicity of ALG-AgNPs in THP-1 cells was performed by using an LDH Assay kit (Dojindo). LDH catalyzes dehydrogenation of lactate to pyruvate, thereby reducing NAD to NADH. NADH reduces a water-soluble tetrazolium salt (WST) in the presence of an electron mediator to produce an orange formazan dye. The amount of formazan dye thus formed is proportional to the amount of LDH released into the medium from damaged cells, which is an indication of cytotoxicity. Briefly, 10^3^ THP-1 cells per well were seeded into 96-well plates and incubated at 37°C with 5% CO_2_. After 24 h, the indicated concentrations of ALG-AgNPs were added and the cells incubated for 48 h. After 48 h, working solution was added to each well and incubated for 30 min, and the reactions were terminated by adding stop solution. The absorbance was then measured at 490 nm by a microplate reader.

### Statistical Analysis

Statistical analyses were performed using GraphPad Prism version 7 software (GraphPad Software Inc., San Diego, CA, United States). All values were given as mean ± SEM. The *t*-test (two-tailed) was used to determine the statistical significance of the difference between two groups. For analyzing multiple groups, one-way ANOVA with a multiple comparison test (Tukey, Bonferroni, Newman-Keuls) or two-way ANOVA with Tukey’s multiple comparison test was used and *p* values < 0.05 were considered statistically significant.

## Results

### Preparation and Characterization of ALG-AgNPs

**FIGURE 1:**
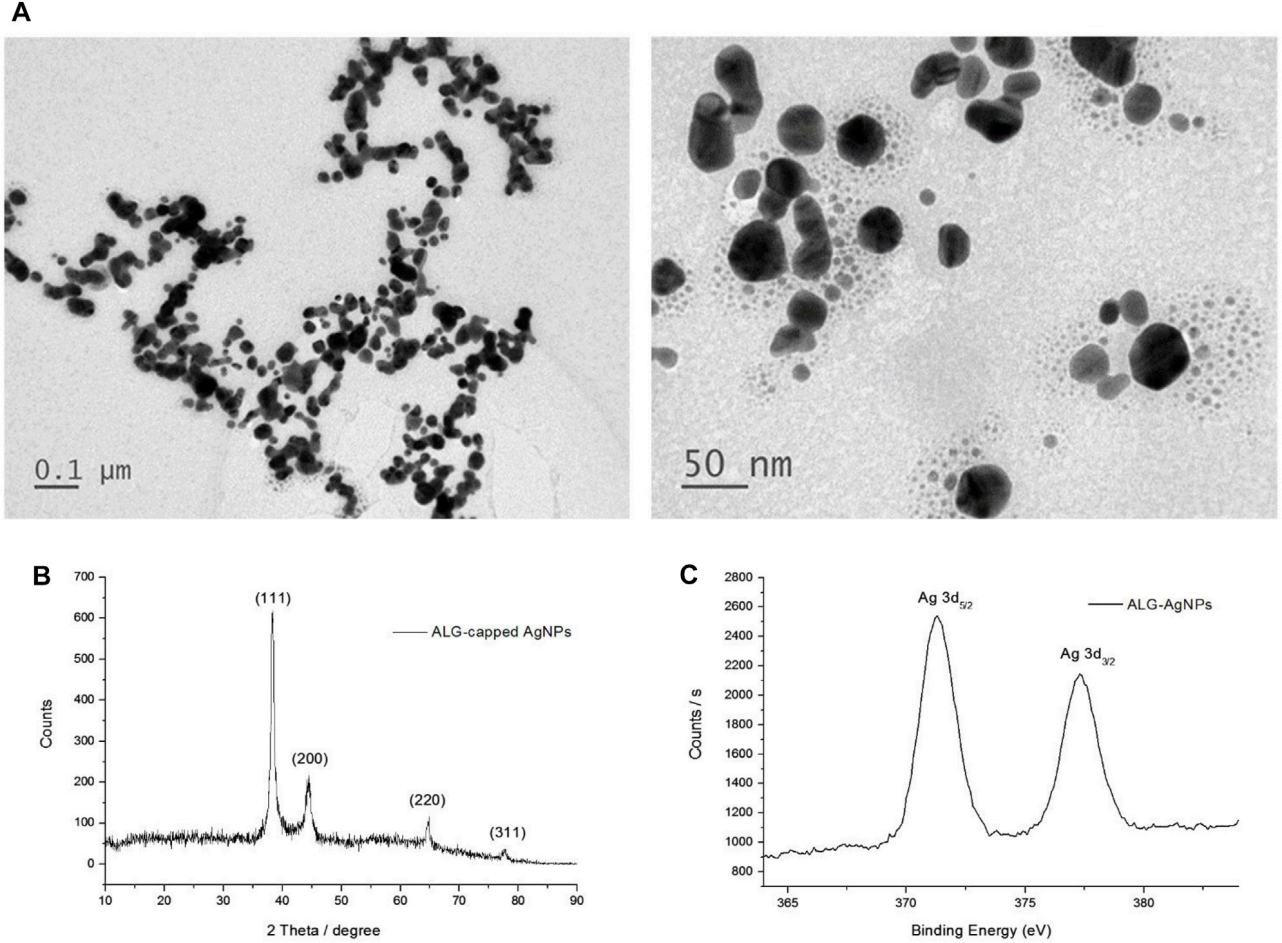
Characterization of ALG-AgNP physical and chemical properties. **(A)** Transmission electron microscopy (TEM) imaging of ALG-AgNPs. **(B)** XRD spectrum of ALG-AgNPs. **(C)** XPS spectrum of ALG-AgNPs.

Our previous report showed that polysaccharides can be used as both a reducing agent and a stabilizing agent to prepare AgNPs (Cheng et al., 2014); however, the reduction reaction progressed very slowly at room temperature. In the present study, we used glucose as a reducing agent, and found that the reaction time could be shortened to 1 h under alkaline conditions. The characteristic SPR peak at around 410 nm confirmed the formation of AgNPs and was also used to estimate the reaction rate of AgNP formation (Chang et al., 2017; Young et al., 2018). The reaction rate, particle size, and zeta potential of the AgNPs were all affected by the concentrations of base, reducing agent, and stabilizer (Supplementary data; Supplementary Figure S1; Supplementary Table S1). Table 1 summarizes the conditions used to synthesize the ALG-AgNPs, as well as the particle size and zeta potential of the as-prepared and dialyzed colloidal solutions. The average particle size and zeta potential used in the following *in vitro* and *in vivo* studies were 70 nm and -47 mV, respectively. The concentration of Ag^0^ was 396 μg/ml, as measured by ICP-Mass, which is smaller than the calculated concentration (539.3 ppm) because the conversion yield is not 100% conversion yield. TEM images showed the purified ALG-AgNPs to be well dispersed, well-defined, and round in shape (Figure 1A). As per our previous observation, the particle size measured using TEM (mostly <50 nm) was smaller than the Z-average diameter (∼70 nm) measured by DLS (39, 40, 42). The X-ray diffraction (XRD) spectrum (Figure 1B) ) of lyophilized colloid sample showed four peaks at 2θ values of 38.1, 44.2, 64.8, and 77.8 deg, corresponding to (111) (200), (220), and (311) reflections, which are consistent with the standard JCPDS database for AgNPs (No. 04-0783). Elementary XPS analysis indicated two peaks located at binding energies of 372.8 and 378.8 eV with a spin-orbit separation of 6.0 eV, which correspond to the emission of the 3d photoelectrons of Ag 3d_5/2_ and Ag 3d_3/2_ (Figure 1C).

ALG-AgNPs have potential anti-mycobacterial activity against various pathogenic strains of *Mycobacterium tuberculosis in vitro*.

**FIGURE 2:**
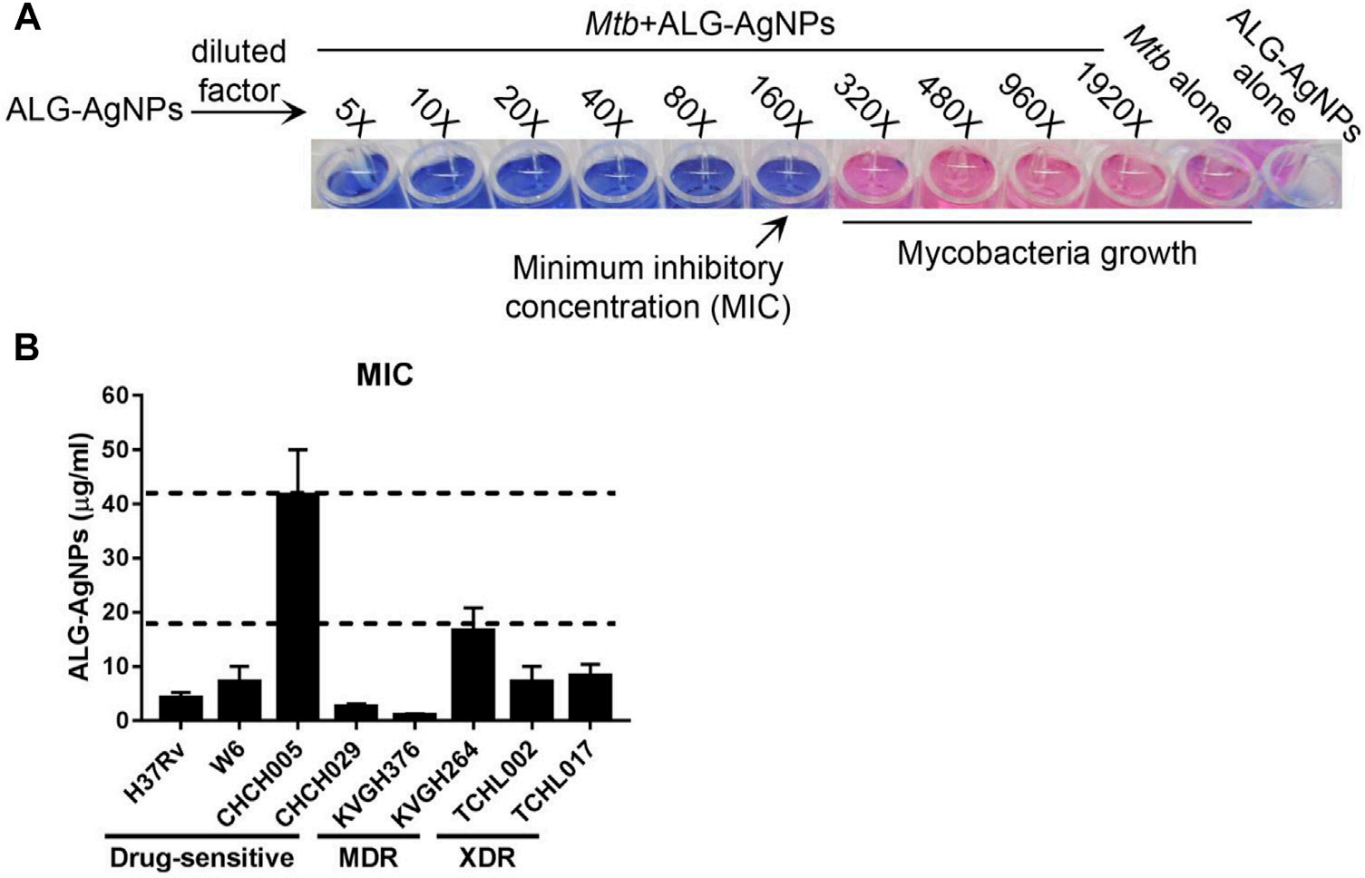
ALG-AgNPs have potential anti-mycobacterium activity against various pathogenic strains of *Mycobacterium tuberculosis in vitro*. **(A)** Schematic diagram of the Alamar blue assay employed for MIC determination. **(B)** MICs of ALG-AgNPs determined for different strains of *Mtb*. The data represent mean ± SEM of 3 experiments. MIC: minimum inhibitory concentration; MDR: multidrug-resistant TB; XDR: extensively drug-resistant TB.

According to epidemiological surveillance data, the Beijing strain of *Mtb* is endemic throughout Asia as well as other parts of the world, and is often associated with highly virulent, multiple-drug resistance (Liao et al., 2015). The East African–Indian (EAI) lineage is prevalent in many tropical Asian countries (Liao et al., 2015; Wada et al., 2017). To determine the anti-mycobacterial activity of ALG-AgNPs against various pathogenic *Mtb* strains, we tested 7 clinical isolates of *Mtb*—two Beijing strains (W6, CHCH005), one EAI strain (CHCH029), two MDR strains (KVGH264 and KVGH376), and two XDR strains (TCHL002 and TCHL017)—and one standard reference strain (H37Rv). H37Rv, a pan-susceptible strain, is the quality-control strain used for anti-mycobacterial drug susceptibility testing by most laboratories (Ghiraldi et al., 2011; Zhao et al., 2018). *In vitro* anti-mycobacterial activity of ALG-AgNPs, reported as the minimum inhibitory concentration (MIC), was evaluated by the microplate Alamar blue assay (MABA). Alamar blue (AB) is an oxidation–reduction indicator dye that has been widely employed to evaluate the sensitivity of mycobacteria to anti-mycobacterial drugs (Franzblau et al., 1998). Reduction of AB dye occurs during mycobacterial growth, which converts the dye from blue to pink. Drug-mediated growth inhibition interferes with AB reduction and therefore the development of pink color. Treatment of pathogenic *Mtb* with increasing concentrations of ALG-AgNPs showed dose-dependent inhibition of *Mtb* growth (i.e., blue wells at higher ALG-AgNP concentrations) (Figure 2A). All clinical *Mtb* strains tested, except CHCH005 (Beijing strain), were sensitive to ALG-AgNPs at an effective concentration of <20 μg/ml; CHCH005 was more resistant (∼40 μg/ml) (Figure 2B). Specifically, the MICs of four drug-sensitive strains were 4.17 ± 1.04 μg/ml for H37Rv, 7.29 ± 2.76 μg/ml for W6 (Beijing strain), 41.67 ± 8.33 μg/ml for CHCH005 (Beijing strain), 2.60 ± 0.52 μg/ml for CHCH029 (EAI strain). For the two MDR-TB strains, the MICs were 1.04 ± 0.26 μg/ml for KVGH376 and 16.67 ± 4.17 μg/ml for KVGH264. Finally, for the two XDR-TB strains, the MICs were 7.29 ± 2.76 μg/ml for TCHL002 and 8.33 ± 2.08 μg/ml for TCHL017 (Figure 2B). This result demonstrates the potential anti-mycobacterial effect of ALG-AgNPs against various pathogenic *Mtb* strains.

**FIGURE 3:**
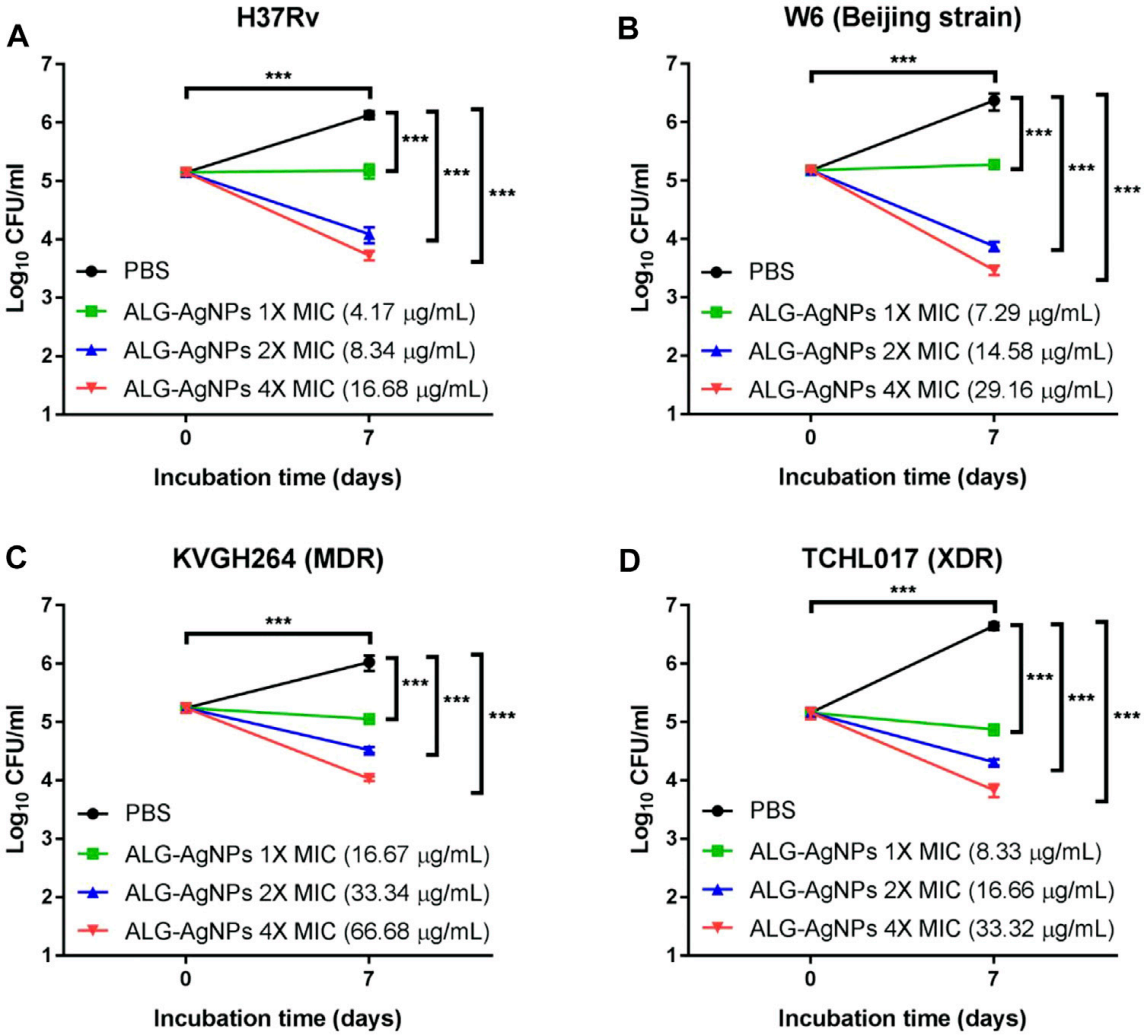
The biocidal action of ALG-AgNPs against different *Mtb* strains measured as a suppression of colony numbers relative to PBS treatment (control). Four *Mtb* strains were incubated with PBS or different amounts of ALG-AgNPs for 0 and 7 days. Bacterial growth was determined by counting the colony forming units (CFU) of **(A)** H37Rv (standard reference strain), **(B)** W6 (Beijing strain), **(C)** KVGH264 (MDR), and **(D)** TCHL017 (XDR). MDR: Multidrug-resistant TB; XDR: extensively drug-resistant TB. The data are expressed as log_10_ CFU/ml and represent the mean ± SEM of 3 experiments. ***p < 0.001.

To confirm the bactericidal activity of ALG-AgNPs against *Mtb,* we evaluated H37Rv, W6 (Beijing strain), KVGH064 (MDR), and TCHL017 (XDR) for enumeration of colony-forming units (CFUs) on 7H10 agar plates after 7 days of treatment with ALG-AgNPs or PBS (control). Colony formation after PBS treatment was significantly increased for all 4 *Mtb* strains compared with day 0 (Figures 3A–D). When treated with 1× MIC of ALG-AgNPs, colony formation of the 4 different *Mtb* strains showed almost no increase at day 7 compared with day 0, indicating that bacterial growth was inhibited by ALG-AgNPs. Of note, the 2-fold and 4-fold MIC treatments caused significant reductions in colony formation of the 4 different *Mtb* strains (Figures 3A–D). These results indicate that ALG-AgNPs have potency against both virulent and drug-resistant *Mtb* strains. Because the MICs for all but one of the strains are less than 20 μg/ml (the exception is < 50 μg/ml) (Figure 2B), we chose ALG-AgNP concentrations of 25, 50, and 100 μg/ml for use in further experiments.

ALG-AgNPs significantly inhibit the growth of cytosolic *Mycobacterium tuberculosis* in a macrophage infection model.

**FIGURE 4:**
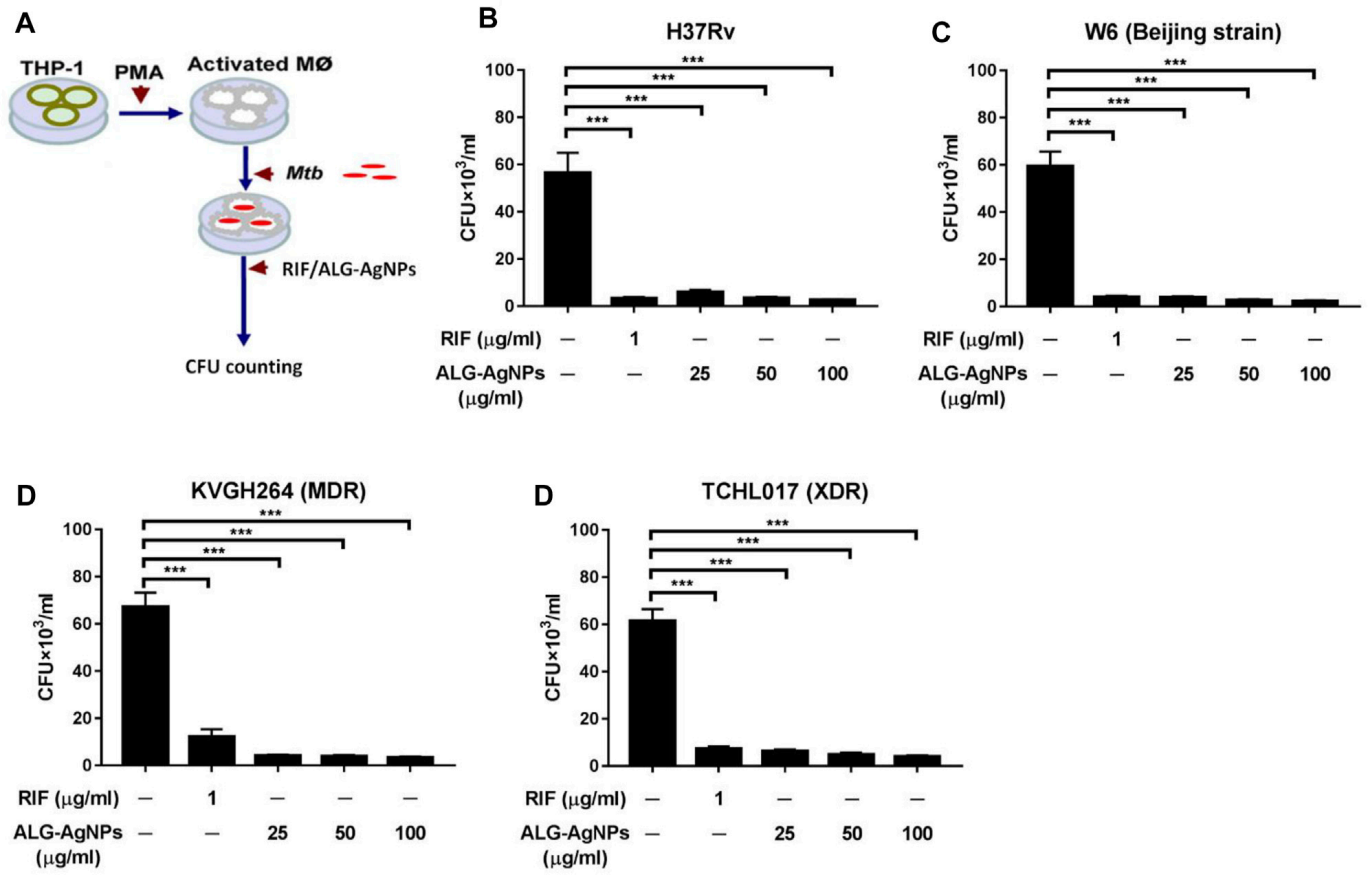
ALG-AgNPs significantly inhibit the growth of cytosolic *Mycobacterium tuberculosis* in a macrophage infection model. **(A)** Schematic diagram of the experimental procedures. **(B–E)** THP-1 cells were differentiated into macrophages by PMA stimulation for 3 days, then infected with different strains of *Mtb* at a MOI of 1 for 24 h. After treatment with PBS, or different amounts of ALG-AgNPs (25, 50, and 100 μg/ml), or rifampicin (RIF) (1 μg/ml) for 5 days, the cells were lysed and plated on 7H10 agar plates. The bacterial growth was determined by CFU of (B) H37Rv, **(C)** W6 (Beijing strain), **(D)** KVGH264 (MDR), and **(E)** TCHL017 (XDR). MDR: Multidrug-resistant TB; XDR: extensively drug-resistant TB. Data represent mean ± SEM of 3 experiments. ***p < 0.001.

*Mtb* is recognized predominantly as an intracellular pathogen. When tubercle bacilli are inhaled into the lungs, they are engulfed by phagocytic cells such as pulmonary macrophages (Guirado et al., 2013; Srivastava et al., 2014). To mimic the behavior of human disease, we used PMA to differentiate THP-1 cells into macrophages *in vitro*, then infected the cells with *Mtb* [H37Rv, W6 (Beijing strain), KVGH264 (MDR), and TCHL017 (XDR)] to mirror *Mtb* internalization. The efficacy of ALG-AgNPs against intracellular *Mtb* was determined by treating *Mtb*-infected macrophages for 5 days with PBS (negative control), RIF (positive control), and increasing concentrations of ALG-AgNPs (25, 50, and 100 μg/ml), then measuring the mycobacterial burden in all infected samples by CFU analysis (Figure 4A). Significantly higher CFU counts were measured in the PBS treatment groups compared with the RIF and ALG-AgNP treatment groups for all four *Mtb* stains (Figures 4B–E). More interestingly, ALG-AgNPs showed good potency in the Beijing- and drug-resistant strains*.* These results confirm the ability of ALG-AgNPs to inhibit intracellular *Mtb*.

### ALG-AgNPs Effectively Suppress the Growth of Dormant-like *Mycobacterium tuberculosis* Bacilli *in vitro*

**FIGURE 5:**
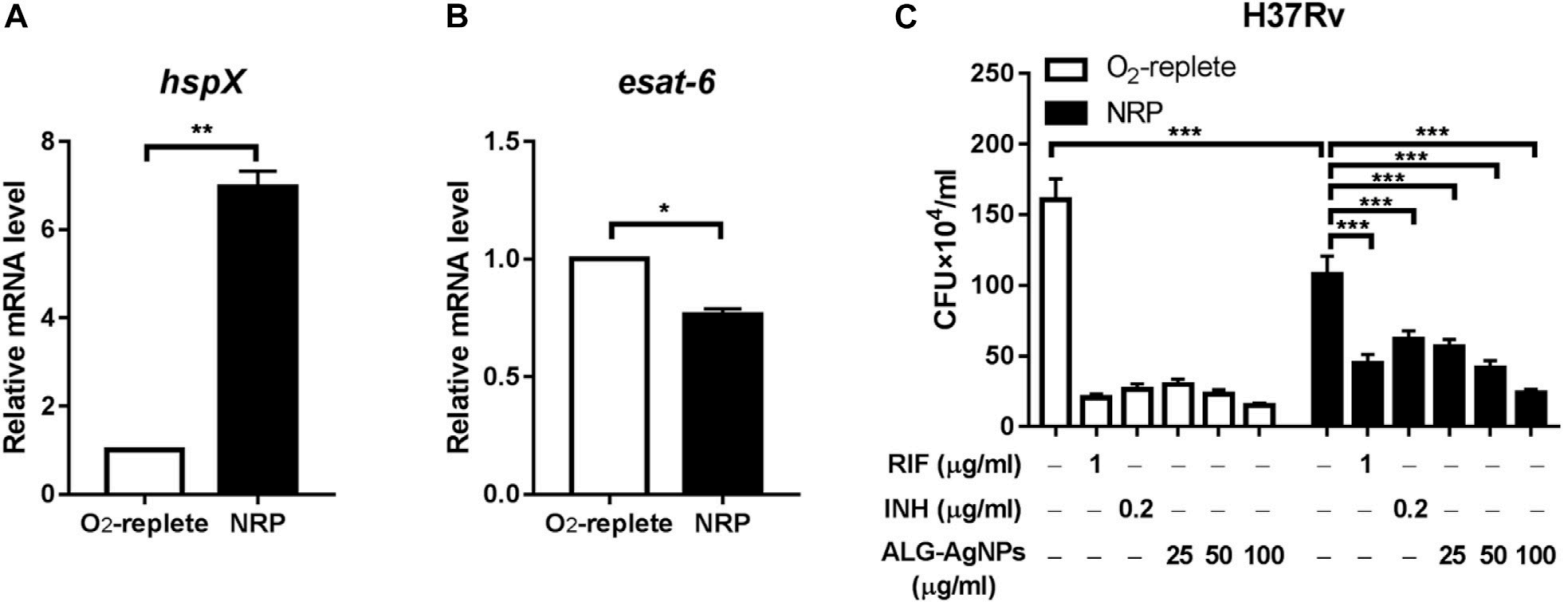
ALG-AgNPs effectively suppressed the growth of dormant-like bacilli *in vitro*. **(A–B)** The *Mtb* strain H37Rv was cultured with or without hypoxia to induce non-replicating persistence (NRP) or not. Then, the gene expression levels associated with the NRP state were assessed by QPCR, specifically **(A)** *hspX* and **(B)** *esat-6*. **(C)** The bacteria were treated with PBS, rifampicin (RIF) (1 μg/ml), isoniazid (INH) (0.2 μg/ml), or different amounts of ALG-AgNPs (25, 50, and 100 μg/ml) in the NRP state or not (O_2_-replete) for 5 days. The bacterial growth was determined by CFU analysis on Middlebrook 7H10 agar and expressed as the number of CFU × 10^4^/ml. NRP: non-replicating persistence. RIF: rifampicin. INH: isoniazid. Data represent mean ± SEM of 3 experiments. *p < 0.05; **p < 0.01; ***p < 0.001.

Latent TB, also known as non-replicating persistent (NRP) TB, develops when mycobacteria become adapted to the host’s immune system and survive within phagocytic immune cells despite hypoxia, nutrient deficiency, and low pH. These mechanisms subvert the immune system and allow infection without any symptoms for decades (de Martino et al., 2019). Current anti-TB drugs have poor activity against NRP *Mtb*. Thus, we investigated whether ALG-AgNPs have potential sterilizing activity against latent TB, by culturing the *Mtb* strain H37Rv either under oxygen-replete or hypoxic conditions to induce an NRP state. We then treated the cells with PBS, RIF, INH, and different amounts of ALG-AgNPs (25, 50, and 100 μg/ml) and assessed *Mtb* survival by CFU analysis. The NRP state was confirmed by measuring the expression levels of two genes, *hspX*, which is known to be transcribed in stationary-phase *Mtb* (Jee et al., 2018), and *esat-6*, which is known to be downregulated when *Mtb* cells enter dormancy (Arroyo et al., 2018). The results of quantitative polymerase chain reaction (QPCR) analysis showed that hypoxic stress significantly induced *hspX* mRNA expression (Figure 5A), whereas the expression of *esat-6* was downregulated (Figure 5B). These data indicate that *Mtb* H37Rv had entered the NRP state in response to hypoxia. Moreover, CFU analysis showed significantly fewer colonies in the NRP state compared with the oxygen-replete condition after PBS treatment, which further confirmed the entry of H37Rv cells into dormancy (Figure 5C). Treatment with RIF and INH, which is used for preventive therapy in LTBI (Villa et al., 2019), produced significant reductions of the bacterial burden in both the oxygen-replete and hypoxia-induced NRP states. More importantly, treatment with ALG-AgNPs produced dose-dependent reductions in CFU counts of NRP H37Rv (Figure 5C). These results indicate that ALG-AgNPs have potential as a preventive therapy against *Mtb* reactivation by suppression of dormant bacilli during latent infection.

### ALG-AgNPs Provide a Therapeutic Mechanism of Biocidal Action Against *Mycobacterium tuberculosis* by Increasing Mycobacterial Cell-Wall Permeability

**FIGURE 6:**
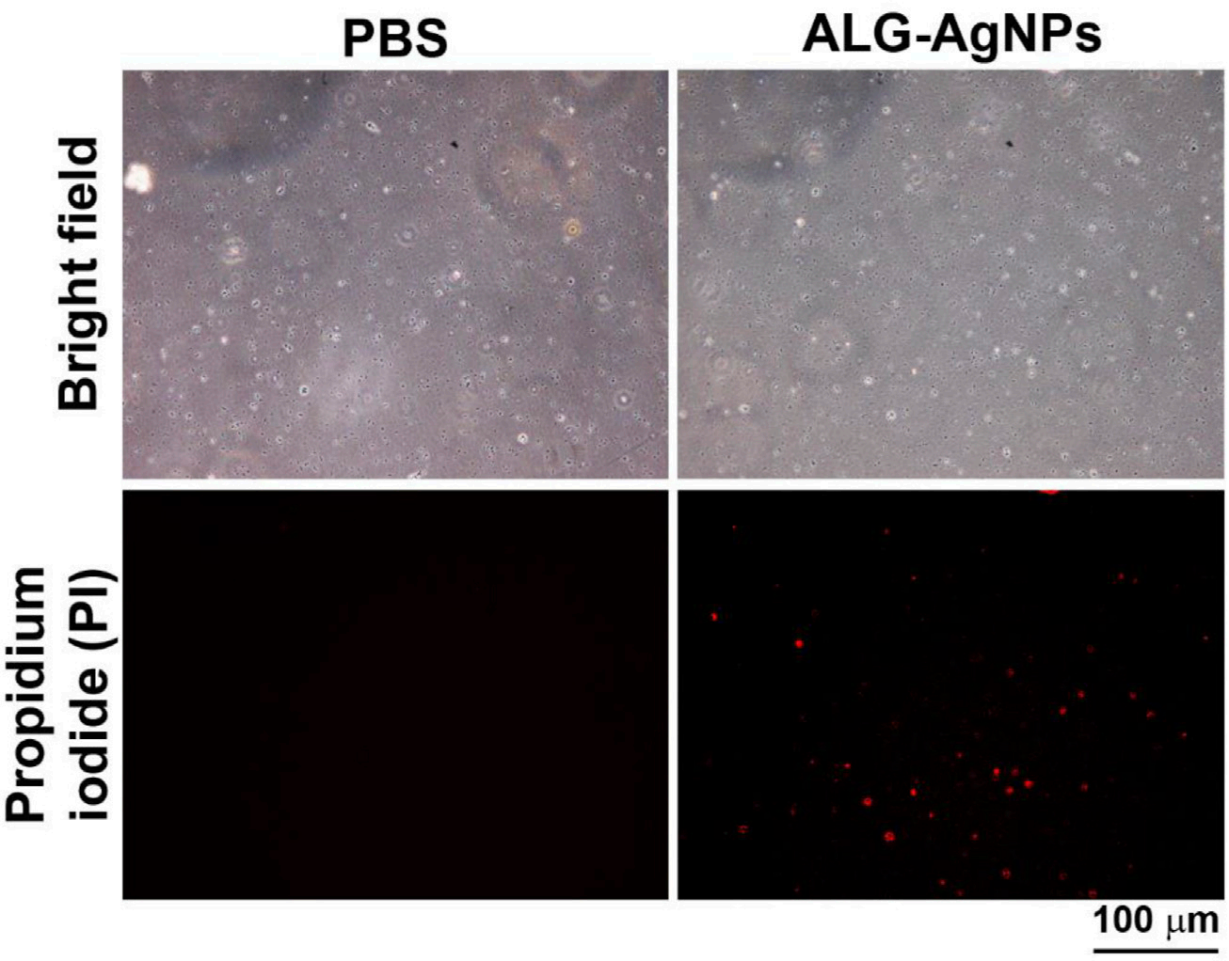
ALG-AgNPs exert their biocidal action against *Mycobacterium tuberculosis via* an increase in bacterial cell-wall permeability. Log phase *Mtb* H37Ra cells were treated with or without 50 μg/ml ALG-AgNPs for 24 h. Then, the bacteria were stained with propidium iodide. The images were observed at 400× magnification by fluorescence microscopy. Scale bar represents 100 μm.

Previous studies have reported that the bactericidal activity of AgNPs is mediated by damage to bacterial cell walls *via* the release of silver ions that produce reactive oxygen species (ROS) to form a redox reaction (Dakal et al., 2016; Yin et al., 2020). Thus, we wondered whether our anti-mycobacterial ALG-AgNPs adopt this mechanism against *Mtb*. To test this possibility, we employed a bacterial cell-wall permeability assay using the *Mtb* strain H37Ra (we used this avirulent strain rather than H37Rv because our P2^+^ facility is not set up for fluorescence microscopy). We treated the cells with or without ALG-AgNPs at 50 μg/ml, which is the concentration that successfully suppressed the growth of the replicating-active drug-sensitive strains H37Rv and W6, the MDR strain KVGH264, and the XDR strain TCHL017 (Figures 4B–E) as well as the dormant *Mtb* strain H37Rv (Figure 5C) without inducing cytotoxicity in THP-1 cells [as measured by lactate dehydrogenase (LDH) released into culture supernatant from damaged or apoptotic cells (Supplementary Figure S3A)]. The bacterial cell-wall permeability assay showed that H37Ra bacilli treated with ALG-AgNPs had greatly increased permeability to the cell-wall-impermeable nucleic acid-binding dye propidium iodide, compared to cells treated with PBS (Figure 6). These results indicate that the anti-mycobacterial mechanism of ALG-AgNPs is related to disrupting the cell wall directly.

### ALG-AgNPs Are a Safe and Effective Therapeutic in Zebrafish and Mouse Tuberculosis Animal Models

**FIGURE 7:**
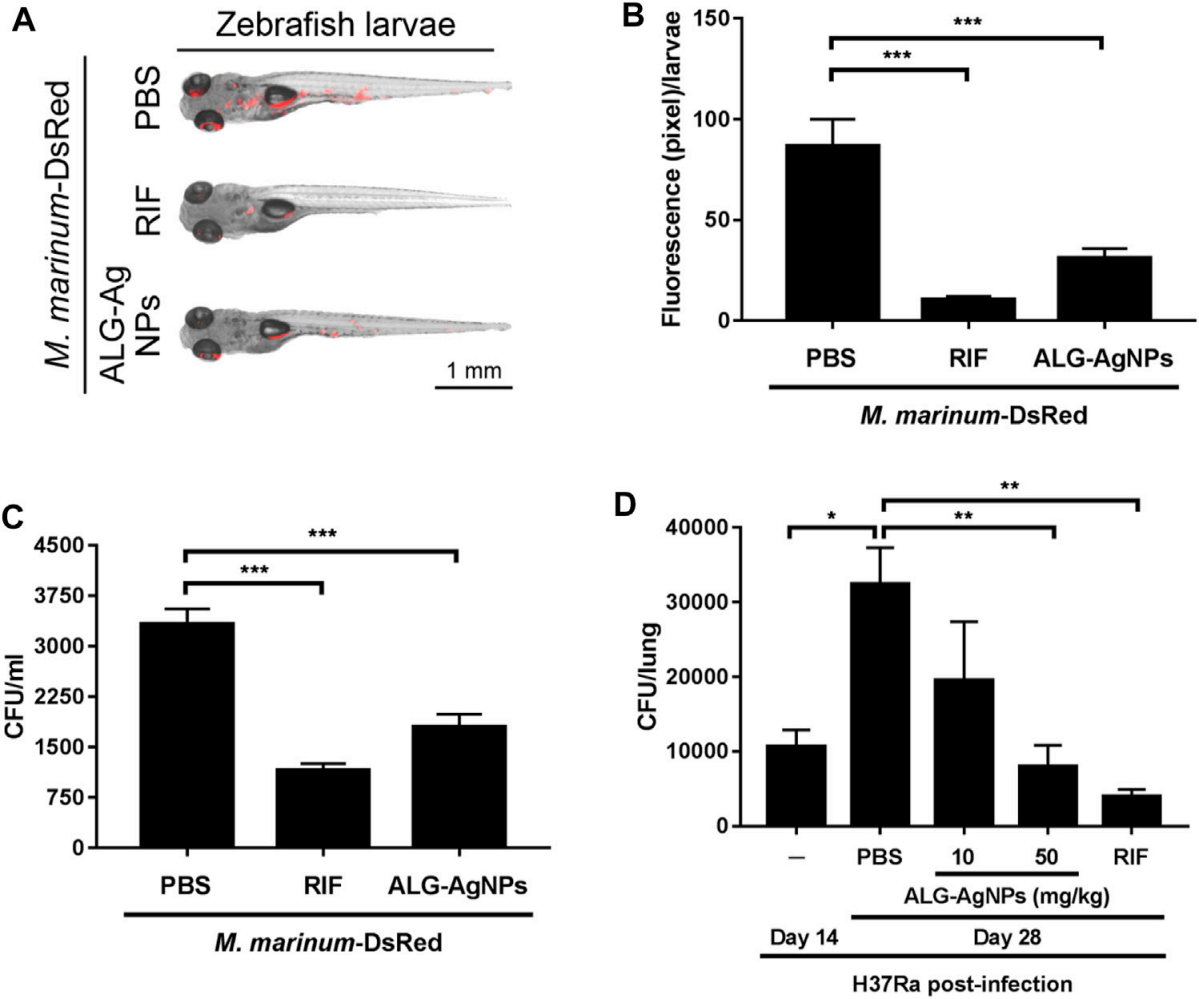
ALG-AgNPs are a safe and effective therapeutic drug in zebrafish and mouse TB animal models. **(A–C)** After microinjection of *M. marinum*-DsRed into the caudal vein of zebrafish larvae, the infected larvae (5 fish/group/experiment) were treated with PBS, 100 μg/ml rifampicin (RIF) or 200 μg/ml ALG-AgNPs for 5 days. All data for zebrafish were from two experiments. **(A)** At 5 days post infection (dpi), the red fluorescence signal from *M. marinum*-DsRed infection was observed by fluorescence microscopy. Scale bar represents 1 mm. **(B)** The fluorescence signals of *M. marinum*-DsRed from zebrafish larvae treated with PBS (*n* = 10), rifampicin (*n* = 10) or ALG-AgNPs (*n* = 10) were quantified using ImageJ software and expressed as fluorescence pixels per larvae. **(C)** For determination of bacterial burdens, five fish/group/experiment were pooled together and homogenized in 1 ml PBS containing 1% SDS. The bacterial burdens of homogenates from infected larvae were diluted 10 × and 100 × in triplicates and enumerated by CFU analysis of two experiments. **(D)** BALB/c mice were intravenously infected with *Mtb* strain H37Ra for 14 days, then treated with PBS (*n* = 5), 10 mg/kg (*n* = 5) or 50 mg/kg (*n* = 5) of ALG-AgNPs, or 10 mg/kg rifampicin (*n* = 5) for another 14 days, then the mice were euthanized. The lungs of treated mice were collected and homogenized. The homogenates were plated on 7H10 agar plates. Bacterial growth in the lungs was determined by CFU analysis. Data in **(B–D)** represent mean ± SEM. *p < 0.05; **p < 0.01; ***p < 0.001.

To confirm the anti-mycobacterial activity of ALG-AgNPs *in vivo*, we used zebrafish larvae as a TB animal model, which we infected with *Mycobacterium marinum*, a strain closely related to human *Mtb* but which in addition expresses DsRed fluorescent protein. We then treated the larvae with PBS, RIF or ALG-AgNPs, and assessed the anti-mycobacterial efficacy by measuring the red fluorescence signals of *M. marinum*-DsRed as well as the bacterial burden from the infected larvae. The images of *M. marinum*-DsRed-infected larvae show a decrease in DsRed fluorescent signal after treatment with either RIF or ALG-AgNPs compared with the PBS control (Figures 7A,B). The CFU analysis similarly demonstrated significant reductions in the mycobacterial burden in RIF- or ALG-AgNP-treated larvae compared with those treated with PBS (Figure 7C).

To examine *Mtb* infection in a mammalian model, BALB/c mice were infected with the *Mtb* strain H37Ra then treated with PBS, RIF, and different amounts of ALG-AgNPs (10, 50 mg/kg). Before the infection experiment, we determined the maximum tolerated dose (MTD) of ALG-AgNPs in these mice by two routes of administration. Mice were given the ALG-AgNPs by oral gavage or intravenous injection once daily for 2 weeks. During this period, mice were observed daily and no significant changes in animal behavior and clear signs of toxicity were found. The mice tolerated a dose up to 500 mg/kg by oral gavage and 250 mg/kg by intravenous injection. Body weight showed an increase of about 8.1% for oral gavage and 6.2% for intravenous administration on day 14 compared with day 1 (Supplementary Figure S3B) and there were no animal deaths (Supplementary Figure S3C). In the mouse TB model, mice treated with PBS showed a significant increase in H37Ra-mycobacterial burden in the lungs on day 28 compared to day 14 (Figure 7D). Importantly, ALG-AgNP treatment dose-dependently decreased the mycobacterial burden in the lungs on par with RIF (Figure 7D). These data demonstrate that ALG-AgNPs are non-toxic *in vivo*, and that their anti-mycobacterial potential is strong in both zebrafish and mouse TB animal models. These results suggest ALG-AgNPs could provide a new therapeutic option to treat *Mtb*.

## Discussion

According to our previous studies, the anti-microorganism activities and cytotoxicity of AgNPs are highly dependent on the capping materials used and the surface charge. For example, chondroitin sulfate-stabilized AgNPs showed good antimicrobial activities against *Acinetobacter baumannii* (including multidrug-resistant strains) and *Pseudomonas aeruginosa* (Young et al., 2018); trimethylchitosan-stabilized AgNPs showed antimicrobial activity against multidrug-resistant *A. baumannii* (Chang et al., 2017) and fungicidal activity against *Candida* species (Wang et al., 2020b). In the present investigation, we utilized eco- and biologically friendly methods to synthesize AgNPs by using alginate as a stabilizer and glucose as a reducing agent. This method was carried out in aqueous solution under ambient conditions without producing any interfering impurities or intermediates, and produced high yields of AgNPs reproducibly. Furthermore, this facile fabrication process does not require complicated instruments and purification processes, thus it can be easily transferred to industrial manufacturing scales in the future. In addition, the average size of our ALG-AgNPs was characterized as nanoscale (Table 1), with a uniform size distribution and high stability (Supplementary Figure S2). Moreover, ALG-AgNPs also showed low cytotoxicity in mouse L929, human MCF-7, human A549, and canine MDCK cells based on an *in vitro* LDH release assay and cell cycle analysis by flow cytometry (40, 41).

In theory, nanoparticles might have greater biocidal capacity than microparticles because a greater number of nanoparticles could interact with the mycobacterial cell wall per the same area. Previous studies support the idea that smaller-size particles exert greater anti-microbial activity than larger particles (Zhang et al., 2016; Yin et al., 2020). The shape of ALG-AgNPs is equally important as a determinant of biocidal activity. Smaller AgNPs with spherical or quasi-spherical shapes are more prone to release silver ions owing to their larger ratio of surface area to volume (Zhang et al., 2016). This also explains the observation that aggregated AgNPs release fewer silver ions compared to well-separated AgNPs. While AgNPs have been utilized previously to treat microbial infections such as *Mtb* (Agarwal et al., 2013; Tăbăran et al., 2020), ours is the first study to use alginate-capped AgNPs and show anti-mycobacterial activity against various pathogenic *Mtb*, including drug-sensitive and drug-resistant strains. These drug-sensitive strains include a standard reference strain (H37Rv), Beijing- (W6 and CHCH005), and EAI (CHCH029) strains. The latter two families of *Mtb* are prevalent worldwide and/or in Asian countries (Liao et al., 2015; Wada et al., 2017). In addition, we also tested four highly aggressive and virulent MDR- (KVGH376 and KVGH264) and XDR- (TCHL002 and TCHL017) strains. The effective concentrations of ALG-AgNPs ranged from 1.04 to 41.67 μg/ml (Figure 2B). Although there are many new anti-TB drugs or derivatives of existing compounds with novel targets in various stages of clinical development or recently approved (Mdluli et al., 2015; Zuniga et al., 2015; Shetye et al., 2020), these drugs target only limited biochemical processes (e.g., protein translation, lipid transport and synthesis, cell-wall biosynthesis, and ATP production). The long-term efficacy of these drugs will be impaired by the potential for cross-resistance. For example, resistance to bedaquiline (BDQ) and delamanid (DLM), the most recently developed and FDA-approved anti-TB drugs, has been reported in clinical settings, giving rise to concern that TB may become an incurable disease (Bloemberg et al., 2015; Singh et al., 2017).

Even though immune responses to *Mtb* are triggered after infection, they are only partially effective and do not reliably eliminate the pathogen. In addition, these immune processes drive the *Mtb* into a latent state of infection, which is reversible when the host immunity becomes weakened (de Martino et al., 2019). Notably, our results showed that ALG-AgNPs can further inhibit cytosolic drug-sensitive- (H37Rv), Beijing- (W6), MDR- (KVGH264), and XDR- (TCHL017) strains of *Mtb* which were engulfed by macrophages (Figure 4), without induction of significant toxicity to THP-1 cells (Supplementary Figure S3A), a cell line derived from human peripheral blood monocyte-like cells. The toxicity of unencapsulated AgNPs has been reported to inhibit the proliferation and migration of mammalian endothelial cells in angiogenesis through the activation of caspase-3 and DNA fragmentation (Kemp et al., 2009). Furthermore, AgNP-treated human cells exhibited various abnormalities, including alterations in cell morphology, decreased cell viability, and increased oxidative stress leading to mitochondrial damage and increased production of ROS, culminating in cell death (AshaRani et al., 2009). The toxicity of AgNPs mainly depends on their physicochemical properties and/or biological coatings on the nanoparticle surface (Zhang et al., 2016). The use of alginate to encapsulate AgNPs was found to be safe to immune cells targeted by *Mtb*.

Nanoparticles and microparticles are selectively taken up *via* phagocytosis by macrophages (Gustafson et al., 2015). This is an important advantage, because these are the very cells where *Mtb* resides. Moreover, these immune cells are actively recruited to the TB granuloma (Ulrichs and Kaufmann, 2006; Ndlovu and Marakalala, 2016), thus delivering the drugs directly to the site of infection. Most importantly, after endocytosis of *Mtb* by immune cells, the pathogen can establish a niche to avoid clearance by the immune system and become dormant-like in cells. In our hypoxia-induced NRP model, ALG-AgNPs inhibited *Mtb* growth dose-dependently during dormancy (Figure 5C), suggesting that ALG-AgNPs might be used to treat latent TB. Although INH and RIF can treat latent TB, MDR-TB is resistant to both INH and RIF, the two most potent TB drugs. In addition, the emergence of drug-resistant TB strains is increasing. The thickened cell wall of *Mtb* blunts the effectiveness of INH and RIF.

Previous findings have indicated several mechanisms of killing of microbes by AgNPs (Qing et al., 2018). Among these mechanisms, AgNPs release substantial amounts of silver ions. The adherence of silver ions to the bacterial cell wall and cytoplasmic membrane disrupts the bacterial envelope (Qing et al., 2018). Indeed, our results from the cell-wall permeability assay revealed that ALG-AgNPs render the cell wall of *Mtb* penetrable and porous (Figure 6), presumably through oxidation-induced permeabilization. The mechanism also involves the formation of ROS, which are induced both by nanoparticles and by silver ions. The treated bacteria could have an increased intracellular concentration of ROS, its toxicity exacerbated by the presence of silver ions. Furthermore, once inside the microbial cell, Ag ions are capable of inhibiting enzymes of the respiratory chain. This results in a blockade of the oxidation and phosphorylation processes in microbial cells, which may cause bacterial cell death (Qing et al., 2018).

An emerging area in anti-TB experimental therapy is to combine metallic nanoparticles with antibiotics to enhance the anti-mycobacterial efficacy *via* increased drug sensitivity (Deng et al., 2016; Farooq et al., 2019; Jyoti et al., 2019), especially in the context of bacterial strains which tolerate antibiotics. AgNPs can permeabilize the cell wall of *Mtb* and thereby further increase the pathogen’s susceptibility to antibiotic treatments. It is postulated that combining AgNPs with antibiotics could synergistically inhibit MDR-TB, and this is a direction worth pursuing in the future. Despite much evidence demonstrating the efficacy of AgNPs to kill several human pathogens by permeabilizing the cell membrane (Dakal et al., 2016; Bondarenko et al., 2018), the therapeutic efficacy and safety of AgNPs against *Mtb in vivo* remains unclear. The therapeutic effects and benefits of ALG-AgNPs against *Mtb in vivo* were further supported in both our zebrafish and mouse TB animal models. After ALG-AgNP treatment in the zebrafish model, the red fluorescence of *M. marinum*-DsRed infection was reduced (Figures 7A,B) and the bacterial burden was significantly decreased compared with the PBS treatment (Figure 7C). In addition, the mouse TB model infected with the human attenuated *Mtb* strain H37Ra provided further evidence that the anti-mycobacterial activity of ALG-AgNPs reduces the bacterial burden in the lungs (Figure 7D) without induction of host lethality (Supplementary Figure S3C) or evident toxicity, as reflected in no body weight loss (Supplementary Figure S3B). Our results show that the use of biocompatible alginate improved not only the excellent sterilizing ability of AgNPs, but also their biocompatibility, and lowered their cytotoxicity, effects which were evident in both the zebrafish and mouse TB animal models.

Our study has some limitations. First, our ABSL3 facility was occupied during the COVID-19 pandemic to focus on coronavirus studies. Second, the lung was firstly analyzed to evaluate the therapeutic effect of ALG-Ags in the mice model. However, the other organs were not analyzed due to facility unavailable. The efficacy and therapeutic effect of ALG-AgNPs in mice will be investigated henceforth.

In conclusion, the recent nanotechnology revolution is providing new and hopeful therapeutic approaches to improve on the current anti-mycobacterial treatments. Investigation of the mechanisms of drugs based on silver nanoparticles is of particular interest (Dakal et al., 2016). It should be noted that silver-based drugs have been used as antiseptic and anti-inflammatory agents for a long time (Politano et al., 2013). Moreover, we used an environmentally compatible and nonpolluting approach to synthesize AgNPs by using alginate as a stabilizing and/or reducing agent in aqueous solution. This synergism of alginate capped-AgNPs for nanoparticle-based therapy for TB may have manifold therapeutic benefits, such as: 1) enhanced duration of drug activity; 2) high carrying capacity for drug delivery; 3) flexibility of versatile routes of administration; 4) feasibility of enclosing numerous drug types into the matrix; and 5) far lesser side effects and better compliance. Thus, our results verify the hypothesis concerning the mechanism of action of ALG-AgNPs, which is associated with both the alginate and silver particles, the combination of the two forming a safe and promising anti-TB agent. ALG-AgNPs offer a new approach that overcomes many of the difficulties of current TB treatments.

## Data Availability

The original contributions presented in the study are included in the article/Supplementary Material, further inquiries can be directed to the corresponding author.
