# Supplementary material for: Alginate-Capped Silver Nanoparticles as a Potent Anti-mycobacterial Agent Against Mycobacterium tuberculosis
Source: Front Pharmacol. 2021 Nov 17;12:746496. doi: 10.3389/fphar.2021.746496 (PMC8660078; doi:10.3389/fphar.2021.746496)
Supplement: Supplementary file 1 [file DataSheet1.PDF]

Supplementary Data

Title: Alginate-capped silver nanoparticles as a potent anti-mycobacterial agent against *Mycobacterium tuberculosis*

Table S1. The effect of the final concentration of glucose, ALG and NaOH on particle size and surface charge of ALG-AgNPs.

Final Concentration				Size*	Zeta potential*
$AgNO_3$ (mM)	Glucose (mM)	ALG (mg/ml)	NaOH (mM)	nm	mV
5	5	5	80	39.8 ± 8.8	-41.1 ±3.9
5	5	5	40	47.4 ± 5.7	-37.0 ±6.4
5	5	5	20	49.3 ± 14.7	-45.9 ±4.6
5	5	5	10	60.6 ± 18.9	-50.4 ±3.4
5	5	5	5	41.4 ± 23.6	-54.5 ±7.1
5	5	5	2.5	89.9 ± 48.2	-53.9 ±4.5
5	10	5	20	74.3 ± 15.4	-53.2±2.2
5	2.5	5	20	62.2 ± 21.7	-55.1 ±2.2
5	1.25	5	20	63.7 ± 17.3	-48.4 ±9.3
5	5	10	20	98.5 ± 29.6	-54.4 ±6.3
5	5	2.5	20	55.1 ± 11.0	-52.1±4.1
5	5	1.25	20	47.0 ± 9.3	-49.9±6.0

* Average size and Zeta potential of 5 time points (1, 3, 5, 24, 48 hours) measured by the DLS method.

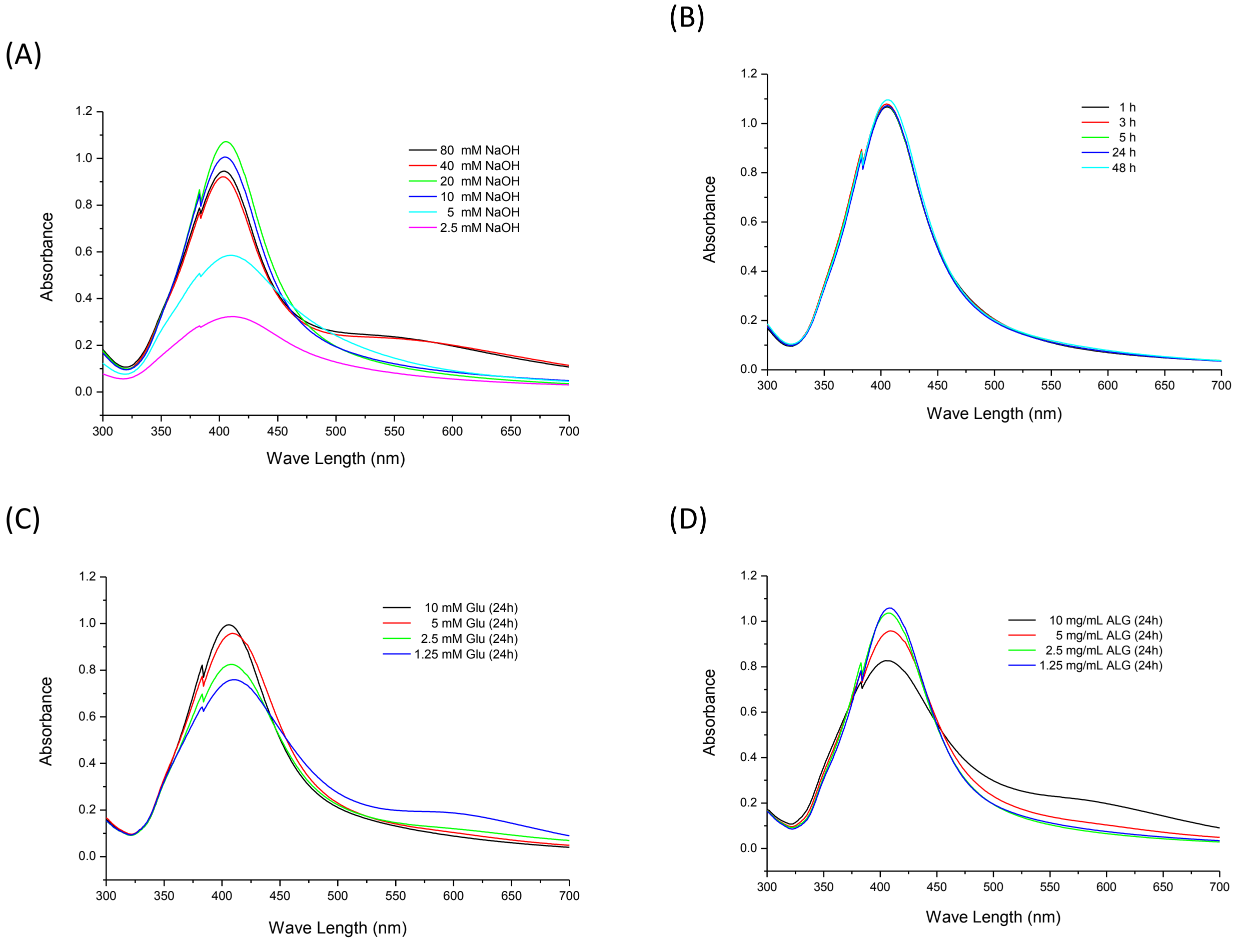

Fig. S1 A~D show SPR peaks of ALG-AgNPs in the different reaction conditions and the corresponding particle sizes and zeta potentials are listed in **Table S1**. To monitor the nucleation and growth of ALG-AgNPs, aliquots of concentrated colloid solution were taken at different time points and diluted 50-fold with Milli-Q water to measure the SPR peak, particle size and surface charge. The characteristic SPR peak at around 410 nm confirmed the formation of AgNPs. The increase in intensities of the SPR peaks indicated the increase in the concentration of AgNPs. Apparently, the reaction rates, sizes, and zeta potentials of AgNPs are affected by the concentrations of the base (NaOH), the reducing agent (glucose), and the stabilizer (alginate). The effect of the final concentration of NaOH, glucose, and alginate (ALG) on the surface plasmon resonance (SPR) peaks of alginate-capped silver nanoparticles (ALG-AgNPs). (A) UV-Vis spectra of ALG-AgNPs synthesized at different NaOH concentrations [$AgNO_3$=5 mM, glucose=5 mM, ALG=5 mg/mL]. (B) UV-Vis spectra of ALG-AgNPs synthesized for different reaction times [$AgNO_3$=5 mM, glucose=5 mM, ALG=5 mg/mL, NaOH=20 mM]. (C) UV-Vis spectra of ALG-AgNPs synthesized at different glucose concentrations [$AgNO_3$=5 mM, ALG=5 mg/mL, NaOH=20 mM]. (D) UV-Vis spectra of ALG-AgNPs synthesized at different ALG concentrations [$AgNO_3$=5 mM, glucose=5 mM, NaOH=20 mM]. All UV-Vis spectral peaks were measured after 50-fold dilution with Milli-Q water of the as-prepared colloidal solutions.

(A)

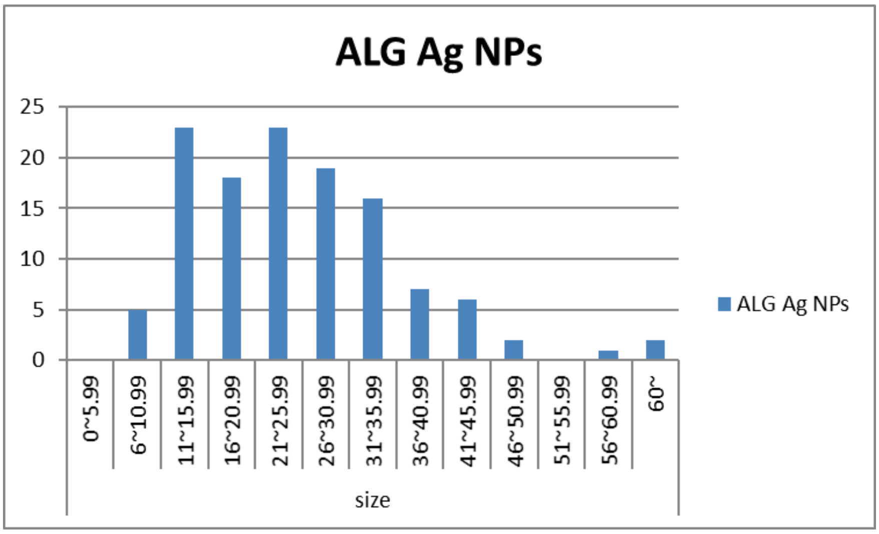

(B)

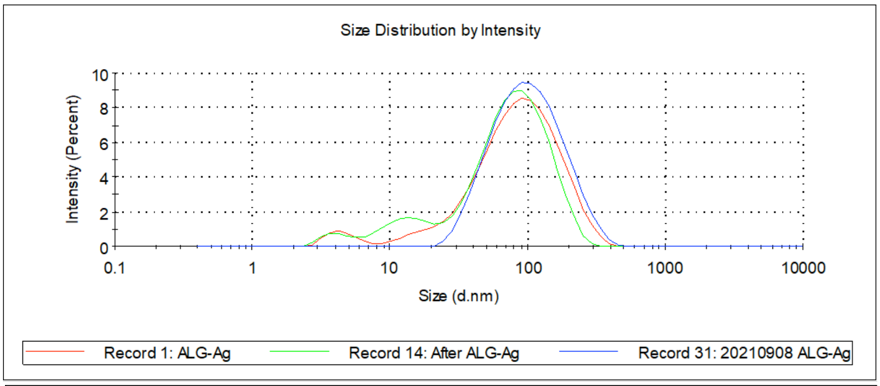

(C)

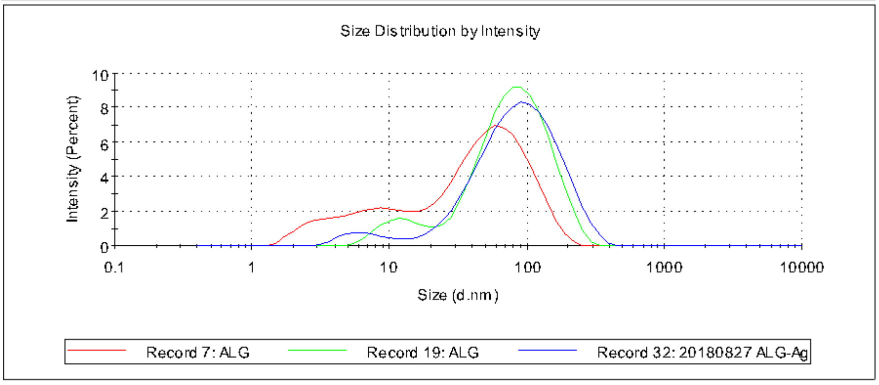

Figure S2. (A) Size distribution of dialyzed ALG-AgNPs measured from TEM. (B) Intensity size distribution of as-prepared (red line), dialyzed (green line), and three-month-stocked (blue line) of ALG-AgNP colloid measured by the DLS method from some batches. (B) Intensity size distribution of as-prepared (red line), dialyzed (green line), and three-year-stocked (blue line) of ALG-AgNP colloid measured by the DLS method from some batches. (B) and (C) were obtained from different batches of synthesis.

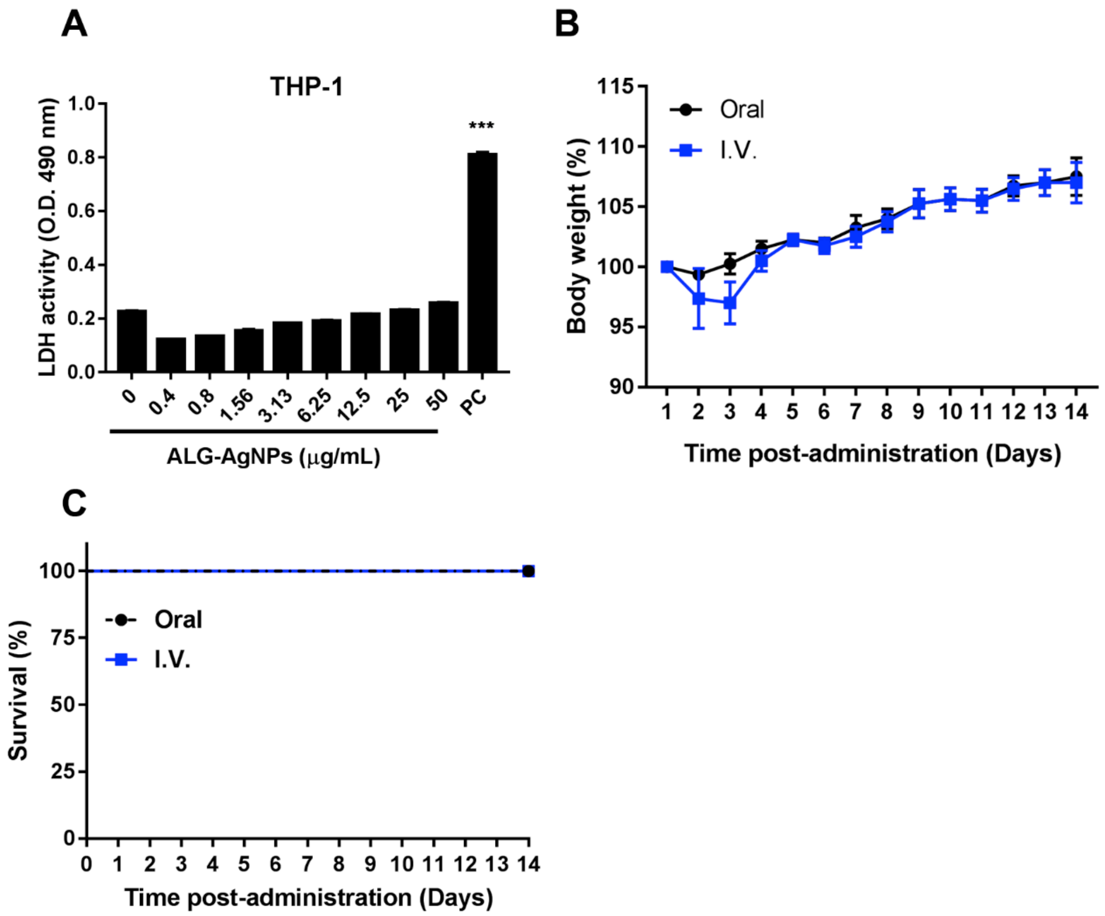

Figure S3. The toxicity and safety of ALG-AgNPs were evaluated in THP-1 cells *in vitro* and in BALB/c mice *in vivo*. (A) THP-1 cells were incubated in 10% fetal bovine serum (FBS) in RPMI with different amounts of ALG-AgNPs for 48 h. The cytotoxicity of the indicated concentrations of ALG-AgNPs in THP-1 cells was measured by LDH activity in the culture supernatants. PC: positive control, THP-1 cells were lysed with 0.1% (v/v) Triton X-100. (B-C) BALB/c mice were administered 500 mg/kg ALG-AgNPs by oral gavage (n = 4) or 250 mg/kg by intravenous injection (n = 4) once daily for 2 weeks. (B) The body weight of treated mice was monitored daily. For the change of body weight as a percentage (%), the body weight at day n was divided by the weight at day 1 for each mouse and multiplied by 100. (C) Mouse survival was monitored daily and analyzed by the Kaplan-Meier method. Data in A-B represent mean ± SEM. ***P < 0.001.
